# Geneticin Stabilizes the Open Conformation of the 5′ Region of Hepatitis C Virus RNA and Inhibits Viral Replication

**DOI:** 10.1128/AAC.02511-15

**Published:** 2016-01-29

**Authors:** Ascensión Ariza-Mateos, Rosa Díaz-Toledano, Timothy M. Block, Samuel Prieto-Vega, Alex Birk, Jordi Gómez

**Affiliations:** a Instituto de Parasitología y Biomedicina López-Neyra CSIC, Granada, Spain; b Baruch S. Blumberg Institute, Doylestown, Pennsylvania, USA; c Department of Pharmacology, Weill Medical College of Cornell University, New York, New York, USA; d CIBERehd Centro de Investigación Biomédica en RED de Enfermedades Hepáticas y Digestivas (ISCIII), Madrid, Spain

## Abstract

The aminoglycoside Geneticin (G418) is known to inhibit cell culture proliferation, via virus-specific mechanisms, of two different virus genera from the family Flaviviridae. Here, we tried to determine whether Geneticin can selectively alter the switching of the nucleotide 1 to 570 RNA region of hepatitis C virus (HCV) and, if so, whether this inhibits viral growth. Two structure-dependent RNases known to specifically cleave HCV RNA were tested in the presence or absence of the drug. One was the Synechocystis sp. RNase P ribozyme, which cleaves the tRNA-like domain around the AUG start codon under high-salt buffer conditions; the second was Escherichia coli RNase III, which recognizes a double-helical RNA switch element that changes the internal ribosome entry site (IRES) from a closed (C) conformation to an open (O) one. While the drug did not affect RNase P activity, it did inhibit RNase III in the micromolar range. Kinetic studies indicated that the drug favors the switch from the C to the O conformation of the IRES by stabilizing the distal double-stranded element and inhibiting further processing of the O form. We demonstrate that, because the RNA in this region is highly conserved and essential for virus survival, Geneticin inhibits HCV Jc1 NS3 expression, the release of the viral genomic RNA, and the propagation of HCV in Huh 7.5 cells. Our study highlights the crucial role of riboswitches in HCV replication and suggests the therapeutic potential of viral-RNA-targeted antivirals.

## INTRODUCTION

Chronic hepatitis C virus (HCV) infection is a progressive disease affecting an estimated 185 million people worldwide (1). Several treatments and combination therapies for chronic hepatitis C have gradually been replaced over the last 35 years. The initial treatments, with low efficacy, high costs, and severe side effects, have evolved into today's modern therapies involving direct-acting antiviral (DAA) inhibitors (1). The development of the viral nonstructural protein 5B (NS5B) polymerase inhibitor known as sofosbuvir represents an important advance in the fight against HCV (2, 3). Using sofosbuvir in combination with ribavirin in patients with genotype 3 infection, high rates of sustained virologic response have been obtained, between 68% and 91% in the presence or absence of cirrhosis, respectively (4). While this is a very encouraging result, significant disadvantages still exist: current antiviral treatment options are expensive (1), antiviral resistance is likely to develop (5, 6), there is naturally occurring polymorphism (7, 8, 9), and efficacy is still limited in those patients in whom infection has led to cirrhosis (4). Therefore, new categories of drugs are needed to supplement or replace existing drug regimens.

Geneticin (also called G418) is an aminoglycoside antibiotic known to be effective against infection by members of the family Flaviviridae: both bovine viral diarrhea virus (BVDV) (10), a member of the genus Pestivirus, and dengue virus, a member of the genus Flavivirus (11). The antiviral mechanism of the drug against these viruses is unknown. However, the inability of Geneticin to inhibit replication in yellow fever virus (YFV) in the same cell where dengue virus is blocked (11) suggests that Geneticin interacts directly with viral RNA. If Geneticin worked on the level of general cellular translation, both viruses would be inhibited. Furthermore, it is known that Geneticin specifically interacts with certain tertiary RNA structures formed from asymmetrical internal loops involving noncanonical base pairs (12), as revealed by its interaction with the A site on bacterial 16S rRNA (13, 14). This ribosomal motif, formed between complementary sequences 1404 to 1410 and 1490 to 1496, participates in an essential RNA switch during translation, which is shunted by the drug, provoking loss of translation fidelity (13). The crystal structure of Geneticin bound to a model RNA fragment containing the A site has provided detailed information about its interaction site. The main conclusion was that, compared to other aminoglycosides, Geneticin offers the ability to accommodate several point mutations associated with resistance or phylogenetic variations (14).

Geneticin is the only cell-permeable aminoglycoside known to date. It has been observed to be one of the least toxic aminoglycosides in animal models, where the aminoglycosides tested, in order of increasing toxicity, were as follows: kanamycin and amikacin < geneticin < neomycin, paromomycin, streptomycin, and tobramycin < gentamicin ≪ hygromycin B (15). The clinical use of Geneticin as an antiparasitic agent has also been proposed (16), and its administration has proven helpful in the treatment of genetic disorders (17).

The basis for evaluating such a compound in a highly variable virus like HCV (18) resides in the concept that it may attack sequences in untranslated regions (UTR), such as the 5′ or 3′ ends, which are far less variable, and that although these regions undergo mutations, their functional structures should be more conserved (19) and therefore susceptible to treatment.

The 5′ UTR of HCV and the first third of its downstream core-coding region, approximately nucleotides (nt) 1 to 600, is the most highly conserved sequence among the different isolates (20, 21). This sequence encodes a high variety of tertiary structures that participate in several essential viral functions, such as initiating translation in viral replication, balancing the proportion entering into translation or replication, and stabilizing the viral genome (22–28). Two of the main structures described along the HCV 5′ nt 1 to 570 sequence are of particular interest for the present work: a tRNA-like domain (29–31) and a double-stranded RNA switch structure (32, 33). These domains were primarily identified by the use of structure-dependent RNases P and III, respectively.

**FIG 1:**
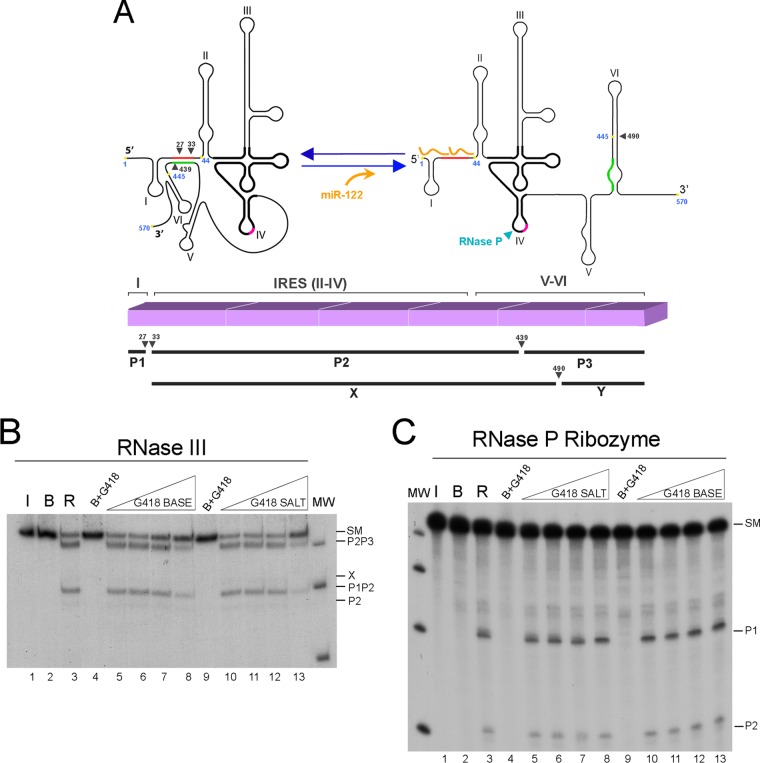
Geneticin's effect on structure-dependent RNases cleaving HCV RNA nt 1 to 570. (A) (Top) Schematic representation of the secondary structure of nt 1 to 570 of HCV RNA open and closed forms and its processing products by RNase III and RNase P. RNase III and RNase P cleavage positions in each of the forms are indicated by black and blue arrowheads, respectively. Highlighted in black is the minimal RNase P recognition site. miR-122 is represented in orange. Long-range annealing sequences are colored red and green. The hypothetical position of Geneticin binding at position 445 is indicated by a yellow spot. (Bottom) Diagram of the sites of cleavage of HCV RNA by E. coli RNase III. Transcript (1–570) is represented by a line. RNase III cleavage sites in positions 27, 33, and 439, respectively, are indicated by three arrowheads. Below are represented the main partial cleavage product bands, P1P2 and P2P3. In the presence of miR-122, a new main cleavage site occurs at position 490, indicative of a different RNA conformation adopted by HCV RNA nt 1 to 570; its main partial products, P1-X and Y, are represented below. (B and C) Determination of the effect of Geneticin on the structural recognition of HCV RNA by RNase III and RNase P ribozymes. Autoradiograms of E. coli RNase III and Synechocystis sp. RNase P ribozyme cleavage of internally labeled RNA with [α-^32^P]GTP (32P RNA) transcripts migrated in 4% polyacrylamide gels for panels B and C. Standard 0.001-U/μl RNase III (B) and RNase P (C) ribozyme reactions were performed on HCV RNA in the absence (lanes 3) of drug or in the presence of increasing concentrations of Geneticin base: 0.5, 5, 50, and 500 μg/ml (lanes 5 to 8) or identical concentrations of Geneticin salt (lanes 10 to 13). Control incubations were done with no enzyme (lanes 2) and with no enzyme but with a Geneticin base (lanes 4 and 9). Lanes 1 were RNA maintained on ice. MW, molecular weight markers.

RNase P is the tRNA precursor (pre-tRNA) processing enzyme that cleaves mature tRNA (34) and structures that mimic tRNA (35–42); these structures include HCV-related animal pestiviruses (43) and unrelated virus families (43–45). Both the RNase P purified from HeLa cells and the ribozyme moiety from the cyanobacterium Synechocystis sp. cleave HCV RNA at a position near the AUG start triplet (Fig. 1A) (29, 30).

RNase III is a specific double-stranded RNase (dsRNase) that cleaves regions of ≥20 bp of RNA in double-stranded structures and certain other specific regions with less perfect base pairings, which invariably contain approximately two turns of essentially regular dsRNA (46–48). Biologically, it participates in several RNA-processing activities, such as the maturation of rRNA (48, 49). A well-studied example is the processing of the early mRNA region, known as R1.1, of the T7 bacteriophage (50, 51). In addition to its primary cleavage activity, RNase III is also known to present secondary cleavage activity (52). The phenomenon was characterized as a set of specific cleavage reactions carried out by the Escherichia coli RNase III enzyme that are favored at high enzyme/substrate ratios and/or higher concentrations of monovalent cations. They behave like primary cleavage reactions because (i) they are effectively inhibited by dsRNA competitors but not by large molar excesses of single-stranded RNA (ssRNA) and (ii) they are reproducible and specific, yielding the same chemical end groups as those of primary cleavages (48, 52).

Several studies indicated that bases 428 to 442 within the HCV coding region interact with the complementary 5′ unstructured sequence, bases 24 to 38, forming a long-range annealing (LRA) motif (closed [C] conformation). Alternatively, the sequence of the core-coding domain may self-fold in what is called stem-loop VI, where bases 428 to 442 are paired with bases 495 to 508 (open [O] conformation). The transition between two double-helical elements has been characterized in the context of a conformational change of HCV RNA nt 1 to 570 (RNA_1–570_) and its dynamic *in vitro* modulation by the liver-specific microRNA. Both dsRNA elements are cleaved by RNase III under secondary conditions of cleavage (32, 33). First, starting in the C conformation, both proximal and distal LRA chains are cleaved, providing P2P3 and P1P2, respectively. After these cleavages, P1P2 is further processed to produce P1 plus P2, and P2P3 either produces P2 plus P3 or switches to spontaneously providing the O form, where stem-loop VI is cleaved to produce a product called X. When HCV RNA nt 1 to 570 is preincubated in the presence of microRNA-122 (miR-122) before cleavage, the microRNA competes with the LRA duplex to bind to HCV sequences between positions 1 and 40 and makes the structure transition to the O form. This form cleaves to produce a product that is longer than X, which was determined to be P1-X, because cleavage at this site is inhibited by the presence of annealed miR-122 (Fig. 1A). RNase III has also been found to cleave BVDV and classical swine fever virus (CSFV) at sites surrounding their respective internal ribosome entry sites (IRESs) (unpublished data). Liver-specific miR-122 has an essential and complex role in HCV biology (25, 26, 53–55).

The main objective of the present work was to discover whether the compound Geneticin, known to bind and perturb RNA structural switches and observed to inhibit the growth of HCV-related virus genera that share common RNA structures in similar regions, can also inhibit HCV. In contrast to work on *Flavivirus* and *Pestivirus*, here, we started by investigating whether Geneticin interfered with RNase III *in vitro* processing and subsequently demonstrated that it could block HCV infection in cell cultures.

## MATERIALS AND METHODS

### Preparation of RNA substrates.

The RNA transcripts used as substrates in the human RNase III assays were derived from plasmid pN(1-4728) Bluescript, which contains nt 1 to 4728 of hepatitis C virus cleaved with BlpI and leaves nt 1 to 570 of the HCV genome under the T7 promoter. Control reactions employed a synthetic DNA containing the sequence of the naturally occurring precursor R1.1 from the T7 bacteriophage, also under the T7 promoter. To obtain the radioactive substrates, 1 to 2 μg of DNA template was transcribed *in vitro* (1 h at 37°C) with [α-^32^P]GTP, followed by a 10-min treatment with RNase-free RQ1 (RNA-qualified) RNase-free DNase I at 37°C and phenol extraction. We used CF11 (cellulose fiber) chromatography to eliminate DNA fragments and nonincorporated nucleotides. The transcripts were then purified using gel electrophoresis under denaturing conditions on 4% polyacrylamide gels containing 7 M urea. The bands were visualized by autoradiography, excised from the gel, and eluted in buffer (100 mM Tris-HCl, pH 7.5, and 10 mM EDTA, pH 7.5). The concentration of radioactive transcripts was determined by calculating the amount of incorporated [α-^32^P]GTP, based on scintillation counting.

### RNase P ribozyme cleavage.

The RNase P ribozyme from strain PCC6803 from Synechocystis species (56, 57) was prepared by *in vitro* transcription as described previously (30). The salt and buffer conditions used for experiments on HCV RNA cleavage were the same as those used previously to detect cleavage on HCV RNA: 50 mM Tris-HCl, pH 7.5, 100 mM MgCl_2_, and 1 M KCl preincubated for 15 min at 37°C before adding the labeled substrate (1.8 nM). The cleavage reaction was performed for 30 min.

### RNase III cleavage.

RNase III digestion was performed on renatured HCV RNA under “secondary conditions of cleavage”: HCV and R1.1 RNA substrates (final concentration, 0.6 nM) were preheated at 90°C for 1 min before adding reaction buffer [10 mM HEPES-KOH, pH 7.5, 10 mM Mg(OAc)_2_ (magnesium acetate), and 100 mM NH_4_(OAc) (ammonium acetate)] and then leaving it to cool to room temperature. In reactions with Geneticin, the drug was added after the 1-hour-long RNA-renaturing step. Cleavage reactions were performed at 0.001 and 0.005 U/ml of E. coli RNase III (Ambion) in the presence of 2 μg of Saccharomyces cerevisiae tRNA and 20 U RNasin (Promega) and were carried out for 1 h at 37°C in a volume of 10 μl. These conditions were used in all of the experiments. The cleavage products were separated on 4% and 10% denaturing polyacrylamide gels for HCV or R1.1 RNA, respectively, and visualized using autoradiography on RX film (Curix RP2 plus; Agfa). Quantitative data regarding RNase III cleavage kinetics were obtained using a phosphorimager (Storm 820; GE Healthcare) scanner and quantified with ImageQuant 5.2 software (GE Healthcare). The percentage of uncleaved material was calculated from the ratio of uncleaved material to starting material (SM).

### Cell culture and virus.

Huh7.5 cells were maintained in Dulbecco's modified minimal Eagle medium (DMEM) (Invitrogen, Carlsbad, CA) supplemented with 10% fetal bovine serum (3). A plasmid containing the full-length HCV Jc1 cDNA was cloned from chemically synthesized DNA oligomers in collaboration with Ju-Tao Guo and Jinhong Chang (Baruch S. Blumberg Institute, Doylestown, PA, USA). Infectious HCV Jc1 was obtained by transcribing HCV Jc1 RNA *in vitro* using the Megascript kit (Ambion), followed by electroporation into Huh7.5 cells and collection of infectious viral particles from the cell culture (58). HCV RNA was transcribed *in vitro* using the Megascript kit (Ambion) and electroporated into Huh7.5 cells (59). Generation of a virus stock and determination of virus titers (50% tissue culture infective doses [TCID_50_] per milliliter) were carried out as described in previous work (60). In general, infection of Huh7.5 cells at a multiplicity of infection (MOI) of 0.015 for 4 days resulted in virus yields of 0.5 × 10^4^ to 0.5 × 10^5^ TCID_50_/ml (61).

### Immunofluorescence detection of HCV infection.

Four days postinfection with HCV Jc1 (MOI = 0.015), HCV Jc1-infected cells were fixed with phosphate-buffered saline containing 1% paraformaldehyde and then incubated for 20 min at −20°C with cold methanol. The cells were then blocked and incubated with HCV NS3 primary antibody (clone H23; Abcam). Bound primary antibody was detected with Alexa Fluor 488-conjugated goat anti-mouse IgG (Invitrogen) and visualized using a Zeiss fluorescence microscope. The total number of foci was determined, as was the number of stained cells in each focus.

### RNA quantification of secreted HCV Jc1 by qRT-PCR.

Four days postinfection with HCV Jc1 (MOI = 0.015), RNA in the culture medium was extracted using TRIzol reagent (Invitrogen) and reverse transcribed using SuperScript III (Invitrogen). Quantitative reverse transcription (qRT)-PCR was performed using forward and reverse primers (5′-AGCGTTGGGTTGCGAAAG-3′ and 5′-CACTCGCAAGCGCCCT-3′, respectively) and the probe 5′-6-carboxyfluorescein-CCTTGTGGTACTGCCTGA-molecular-groove-binding nonfluorescent quencher-3′ (Applied Biosystems) in an Applied Biosystems 7500 thermal cycler. The standard curve was generated using serial dilutions of *in vitro*-transcribed full-length HCV Jc1 RNA.

### Cell viability and Geneticin toxicity assays.

Huh7.5 cells were plated at a density of 1 × 10^3^ to 2 × 10^3^ cells/well on 96-well plates. Twenty-four hours later, the cells were washed, and medium containing the required concentration of Geneticin was added before incubating the cells for 4 days. Cell viability was then assessed using the resazurin (alamarBlue) indicator dye. Dye conversion was quantitatively analyzed using a fluorescent plate reader with excitation and emission at 550 and 580 nm, respectively. Cell viability was expressed as a percentage of untreated cells.

## RESULTS

### Geneticin specifically inhibits RNase III cleavage of HCV RNA_1–570_.

Initial RNase III and RNase P cleavage experiments involved a 570-base HCV RNA transcript in the closed conformation. The cleavage products of these two reactions have been characterized (32, 33). Figure 1A shows a schematic representation of the nt 1 to 570 RNA fragment that reveals the final reaction products (P1, P2, and P3) and the partial digestion products (P2P3 and P1P2) as characterized for RNase III, located according to their corresponding positions. The RNase P ribozyme cleavage site is also shown. The autoradiograms in lanes 3 of Fig. 1B and C represent RNase III and RNase P ribozyme cleavage reactions, respectively, while the products are identified on the right of the gels. These initial experiments consisted of incubating the same amount of HCV RNA_1–570_ with concentrations of Geneticin base or its sulfate salt increasing from 0.5 to 500 μg/ml in order to inhibit HCV RNA cleavage by RNase III (0.0005 U/μl) or RNase P ribozyme (67.5 nM). These inhibition reactions were performed in the linear range for conversion of HCV RNA_1–570_ into its products. While cleavage by RNase P ribozyme was insensitive to both forms of the drug, the quantities of nt 1 to 570 RNase III cleavage products decreased when both forms of the drug were used at a concentration of 500 μg/ml (Fig. 1, lanes 8 and 13).

### Conformational effect of Geneticin on RNase III cleavage inhibition of HCV RNA_1–570_.

We subsequently tested the effect of Geneticin on HCV RNA_1–570_ in the presence of miR-122, which switches the RNA from a closed to an open conformation, and in the presence of a truncated nt 44 to 570 RNA fragment. The absence of the first 44 nt in the truncated fragment prevents the formation of the closed conformation. Similar reactions were performed on standard specificity-controlled substrates of RNase III known as R1.1 (32, 62) and dsRNA from the yeast L-A virus genome.

### Inhibition of HCV RNA_1–570_ processing at higher RNase III activity.

**FIG 2:**
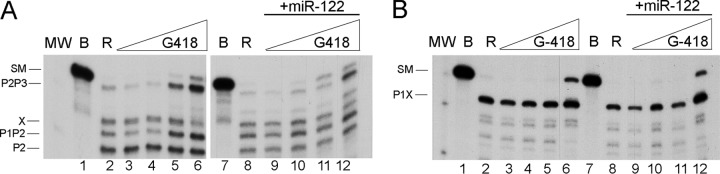
Geneticin inhibits RNase III cleavage of HCV RNA nt 1 to 570 in both the open and closed conformations. (A) Autoradiogram of E. coli RNase III cleavage of internally labeled HCV RNA nt 1 to 570 in the absence (lanes 2 to 6) or presence (lanes 8 to 12) of 15 nM miR-122. Lanes 1 and 7 represent the transcript alone incubated on ice; lanes 2 and 8 represent transcript incubated in buffer and RNase III cleavage at 0.005 U/μl. This standard RNase III reaction, using a constant concentration of HCV RNA, was performed in the presence of increasing concentrations of Geneticin salt: 0.5, 5, 50, and 500 μg/ml (lanes 3 to 6 and 9 to 12). MW, molecular weight markers. The starting material and reaction products are indicated on the left. (B) Same as panel A with the HCV RNA nt 44 to 570 fragment.

In contrast to the experiment illustrated in Fig. 1B, an increased concentration of RNase III was tested to observe, not only the effect of Geneticin on the main HCV RNA partial cleavage products, P1P2 and P2P3, but also the effect on how they are subsequently processed to end products. This is significant, because we previously demonstrated that after the first cleavage, which releases P1 and the partial product P2P3, the latter switches its conformation from a closed to an open form, thus providing the opportunity for a new cleavage in HCV RNA stem-loop VI, at position 490, giving rise to the band product known as X. Figure 2A, left, shows that an increase in P2P3 formation was correlated with a decrease in production of its X product.

Figure 2A reveals that when the substrate, or the SM, entered the reaction in the C form, some substrate remained uncleaved after being reacted with between 50 and 500 μg/ml drug; this was similar to the amount of drug needed to observe substrate accumulation when the reaction was performed at a lower RNase III activity. This indicates that cleavage inhibition is independent of RNase III activity and implies there is a direct interaction between the drug and the substrate. When starting with the substrate partially complexed with miR-122, which is predominantly in the O form, a lower concentration was required to leave some intact substrate: a range of 5 to 50 μg/ml instead of 50 to 500 μg/ml was required, which implies that the O form is more sensitive to the drug than the C form. In both cases, there is a direct inverse relationship between the amount of drug used and the amount of substrate remaining after cleavage reactions on both RNA forms, although it should be pointed out that this is not a dose-response assay because the cleavage reaction is saturated. An analysis of the effect of the drug on the cleavage pattern showed similarity between the two situations—the preferential accumulation of the partial product P2P3.

We subsequently tested the HCV RNA nt 44 to 570 fragment (which adopts only the open conformation [33]) in either the presence or absence of miR-122 (Fig. 2B). Its single cleavage at site 490 produces the product X. In the presence of increasing concentrations of Geneticin, cleavage was inhibited in either the presence or absence of miR-122 (Fig. 2B). This result implies that RNase III cleavage of the open conformation is inhibited by the drug.

**FIG 3:**
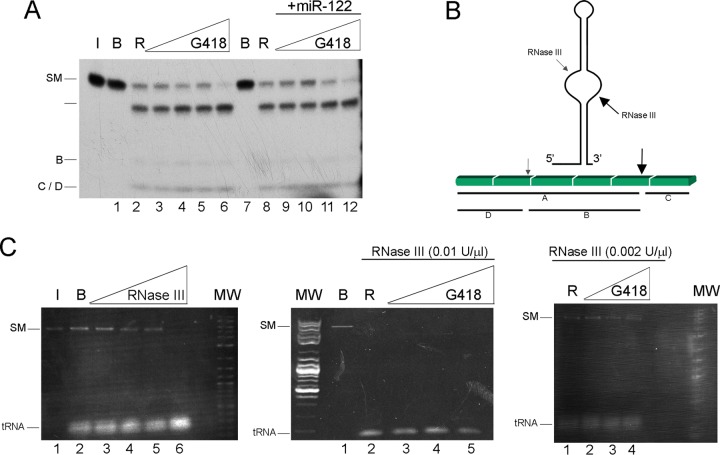
Geneticin does not inhibit RNase III processing of a natural and a standard RNA. (A) Autoradiogram of E. coli RNase III cleavage of control RNA substrate T7 R1.1 mRNA, with the exception that the RNase III concentration was 0.001 U/μl. (B) Schematic representation of the secondary structure of the R1.1 substrate. The primary RNase III cleavage site adjacent to T7 1.1 mRNA is called the “R4 site.” The primary site is well known (depicted in reference 69). Primary cleavage occurs between a U and a G residue of a stem-loop about 20 bp long, with an unpaired “bubble” region in the middle, on the 3′ side of the bubble (large arrow). A secondary cleavage also occurs (62) (small arrow). The primary cleavage always occurs; when the secondary cleavage occurs, it takes place in the larger product of the primary cleavage, yielding two new products. (C) (Left) RNase III activity was calibrated, using a substrate comprising 100 ng per 10 μl of dsRNA of L-A virus extracted from yeast under conditions described previously (70). The cleavage reaction products were run in a 1% agarose gel stained with ethidium. Lane 1, RNA maintained in ice; lane 2, RNA incubated in buffer; lanes 3 to 6, RNA incubated with RNase III activity at 2 × 10^−3^, 5 × 10^3^, 1 × 10^−2^, and 1 × 10^−1^ U/μl, respectively. (Middle) A reaction similar to that for the left gel was performed. Lane 1, L-A virus RNA incubated in buffer; lane 2, L-A virus RNA incubated with RNase III at 0.01 U/μl; lanes 3 to 5, L-A virus RNA in the presence of increasing concentrations of Geneticin (5, 50, and 500 μg/ml). (Right) Same as the middle gel, with RNase III activity of 0.002 U/μl. MW, molecular weight markers corresponding to a log_2_ ladder (NEB).

In order to fully eliminate the possibility that the drug inhibits the enzyme, the effect of Geneticin was tested on the standard substrate R1.1 RNA. The cleavage scheme is shown in Fig. 3B. No inhibition was observed under any of the conditions employed (Fig. 3A); in contrast, subtle cleavage stimulation was observed at the highest drug concentration, a phenomenon that manifested repeatedly. As an additional control, we tested the cleavage of 0.2 μg of perfect dsRNA (obtained from the yeast virus L-A, which has a double-stranded genome) using 0.002 and 0.01 U/ml of RNase III (Fig. 3C). The result ranged from partial cleavage to complete cleavage, independent of the presence of 0.5 to 500 μg/ml of the drug (Fig. 3C).

### Kinetic analysis of the cleavage inhibition mechanism.

**FIG 4:**
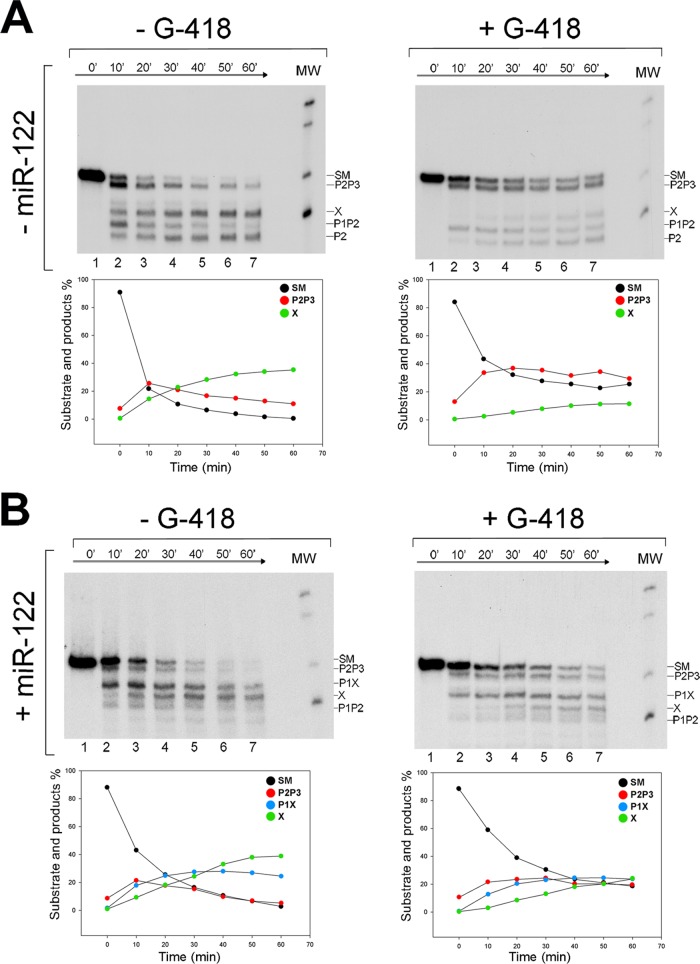
Kinetic analysis of E. coli RNase III cleavage of HCV RNA in the presence or absence of miR-122 and Geneticin. (A) Autoradiogram of RNase III cleavage of internally labeled HCV RNA nt 1 to 570 transcript in the absence (−) or presence (+) of Geneticin. (B) Same as panel A, but in the presence of a synthetic miR-122. Lanes 1 to 7, 0, 10, 20, 30, 40, 50, and 60 min of incubation with RNase III at 0.005 U/μl, respectively. Below each gel is a graphic representation of the time course of processing of SM and main-product generation.

The RNase III kinetic cleavage patterns for experiments in the presence or absence of the drug and in the presence or absence of miR-122 are shown by the gels in Fig. 4A and B. The results from these gels are summarized in the graphs in Fig. 4A and B as the percent formation of cleavage products P2P3, X (in the absence of miR-122), and also P1X (in the presence of miR-122) as a function of time. HCV RNA_1–570_ alone, i.e., incubated in the absence of Geneticin, displayed the characteristic kinetic cleavage pattern obtained previously (Fig. 3B) (33); however, in the presence of the drug, reduced RNase III cleavage was observed (Fig. 4A). Cleavage inhibition was notable in the 10-min SM→P2P3 and P1P2 reaction but was particularly marked in the processing of P2P3→X (either at 10 min or at the end of the reaction). Notice that while in the absence of Geneticin P2P3 and P1P2 were processed nearly to completion, yielding X and P2 products, in its presence P2P3 was retained as the major partial product in the gel and X was consistently the product with the least production. Drug inhibition kinetics had a quantitatively similar effect on the reactions carried out in the presence of miR-122 (Fig. 4B). The least represented products in the presence of Geneticin were X and P1X. The low proportions of X and P1X can be directly associated with the direct inhibition of cleavage at position 490, as observed for the RNA nt 44 to 570 fragment in Fig. 3B.

A very interesting point arises from the cleavage kinetics; in the absence of miR-122 (Fig. 4A), most of the P2P3 produced is already present at the first time point regardless of Geneticin's presence or absence; then, after this point and during the reaction course, while in the absence of the drug SM continues its processing into P2P3, in the presence of the drug, this step is blocked. This suggests that in the presence of the drug the SM enters into the reaction in two different conformations: closed, which was expected for HCV RNA_1–570_ and can quickly be processed to P2P3, and open, which cannot be processed. In turn, this indicates that the drug favors RNA switching to the O conformation in the absence of miR-122. Addition of miR-122 to the reaction mixture did not have a significant effect on the kinetic profiles of the SM and P2P3, based on miR-122 function, except that even less P2P3 was produced by the first time point; thus, miR-122 and Geneticin combined their effects and promoted switching to the open conformation.

### Inhibition of HCV replication in cell culture.

**FIG 5:**
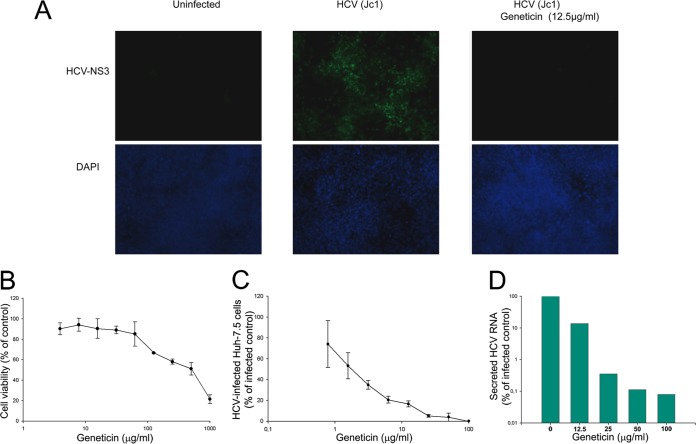
Geneticin inhibits the spread of HCV Jc1 infection in Huh7.5 cells. (A) (Top) Representative visualization of HCV NS3 protein in Huh7.5 cells after 4 days of infection in the presence or absence of 12.5 μg/ml Geneticin. (Bottom) The background cell density was visualized using DAPI (4′,6-diamidino-2-phenylindole). (B) Cell viability in the presence of Geneticin. (C) NS3-stained cells in each focus were counted and quantified for spread of viral infection in the absence and presence of different concentrations of Geneticin. The values represent average results obtained from three independent experiments with EC_50_s of ∼2 μg/ml. (D) Virus yields in culture media as detected by qRT-PCR of culture medium at 96 h postinfection and then normalized to a percentage of the infected control (EC_50_s, ∼12 μg/ml). The values represent averages and standard deviations of results from three independent experiments. The toxicity of Geneticin in Huh7.5 cells was detected using the alamarBlue assay (see Materials and Methods) (LD_50_, ∼530 μg/ml).

To investigate the antiviral effect of Geneticin on HCV replication and viral spread, we first tested Geneticin activity on the initial spread of infection and the formation of HCV Jc1 NS3-positive foci on a monolayer of Huh7.5 cells. The spread of HCV infection in Huh7.5 cells was clearly evident, with well-defined HCV NS3-positive focus formation (Fig. 5A). Significantly, Geneticin diminished viral infection and spread from cell to cell with a 50% effective concentration (EC_50_) of about 2 μg/ml (Fig. 5C and D). Accordingly, the HCV RNA yield in the culture medium was reduced by 10-fold at 12.5 μg/ml and by more than 100-fold at 25 μg/ml (Fig. 5D). Alpha interferon (IFN-α) was used as a positive control, with an EC_50_ of about 10 IU/ml (data not shown). Importantly, the toxicity (50% lethal dose [LD_50_]) of Geneticin in Huh7.5 cells was estimated to be about 530 μg/ml (Fig. 5B), suggesting that the selectivity index (LD_50_/EC_50_ ratio) was about 265, implying selective anti-HCV activity of Geneticin.

## DISCUSSION

The aminoglycoside Geneticin, known for some time to interact with the 16S rRNA of the E. coli ribosome, was applied to RNase P and RNase III cleavage reactions in the HCV RNA_1–570_ region. We observed that while the RNase P ribozyme from Synechocystis sp. is completely insensitive to the drug, it can selectively inhibit RNase III cleavage *in vitro*.

Targeting of small-molecule RNAs has received considerable interest since the discovery that specific interactions between low-molecular-weight metabolites and mRNAs form part of the mechanism controlling the appropriate biological functions of the RNA molecules. These specific interactions are known as riboswitches (63). While there are several screening methods for detection of aminoglycosides binding to RNA (64), our evaluation does not represent a direct analysis of the drug binding to the RNA molecule. Our assay relies on the analysis of the specific structure-dependent relationship of the RNase III-HCV RNA interaction in the presence or absence of the drug. Important factors in assay development included the components involved in the reaction: the drug, the quality of the RNA substrate, the nature of the reaction, and its control. Two forms of the drug, a sulfate salt and a base, were tested in the first experiment and gave similar results, suggesting that the specific structure of Geneticin was responsible for the HCV RNA interaction and that sulfate salt did not influence it. The double-stranded character of the HCV regions being tested in the RNase III cleavage assay might be very sensitive to competition from dsRNA chains generated artifactually during the T7-directed *in vitro* transcription process of the radiolabeled RNA substrate (65). To avoid this, we subjected the transcribed RNA to fractionation on a cellulose phosphate column, capable of separating ssRNA from dsRNA, followed by denaturing electrophoresis, as described in Materials and Methods. The RNase III used was a commercial preparation from a recombinant source that we had previously characterized and validated for its specificity versus authentic E. coli RNase III on T7 R1.1 mRNA, in order to define the HCV cleavages as secondary (32). Therefore, we used exactly the same reaction conditions in the present study.

We have previously demonstrated that two widely separated sequence elements (proximal and distal regions) are simultaneously involved in the LRA motif, while one or the other is cleaved (32). Cleavage at the LRA was also shown to favor transition of the distal LRA strand to form stem-loop VI, where a new RNase III cleavage occurs (33).

**FIG 6:**
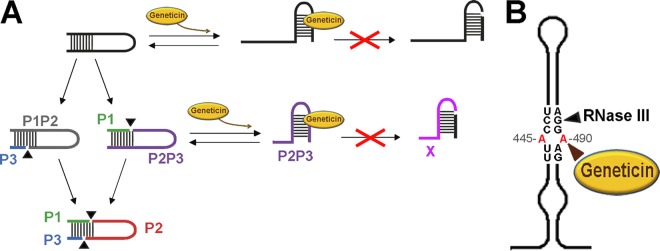
Model of cleavage inhibition and a potential drug binding site. We previously determined that the closed form is cleaved to give band products P2P3 (violet) and P1P2 (gray) (32, 33). Subsequently, each of these products follows a different RNase III processing route. The RNA fragment corresponding to P2P3 switches to an open form of the RNA, forming the stem of domain VI, which is then cleaved to give product band X (pink) (33) (the scheme is shown in Fig. 1A). On the other hand, the P1P2 fragment, still in the closed form, is further processed to provide the P2 (red) fragment. In the presence of miR-122, the fraction of RNA in the open form is cleaved directly to P1X. Other fragments shown are P1 in green and P3 in blue.

At first glance, the fact that cleavage at either the LRA motif in the C conformation (closed) or stem-loop VI in starting material (SM) plus miR-122 (open conformation) was inhibited seems to indicate that the Geneticin-HCV RNA interaction favors a common conformation that cannot be processed by the enzyme. This is supported by kinetic experiments indicating that either the substrate or the larger partial product P2P3 switched to the O form in the presence of the drug. Further processing of the open form to either SM or P2P3 is then inhibited by the drug, as demonstrated by the experiments with the nt 44 to 570 fragment, which is completely open (33), and by the inhibition of P2P3 processing into X. The observation that a lower concentration of drug was required to observe the same effect in the case of SM plus miR-122 than in SM alone also agrees with the inhibition process. In summary, our proposal for the inhibition mechanism is that the drug pays part of the energy cost associated with the transition from the closed to the open conformation, while at the same time, the presence of the drug inhibits RNase III cleavage in the stem-loop VI structure (Fig. 6A).

This mode of action is similar to the mechanism proposed for aminoglycoside-mediated inhibition of the aminoacylation reaction of yeast tRNA^Asp^ (13, 66). The key structure in the ribosomal A site believed to accommodate the aminoglycosides is a dsRNA motif that includes an A · A mismatch adjacent to a bulging adenine (67); interestingly, a similar structure is clearly evident in stem-loop VI (Fig. 6B), positions A_445_ and A_492_, very close to RNase III cleavage at position G_490_.

An alternative hypothesis that cannot be ruled out is that there may be a third stable structure acting as an intermediate in the transition from LRA to stem-loop VI. In fact, experimental evidence for annealing between sequences _372_ACCAAAACG_379_ and _431_CGUUGGU_437_ was gathered using single- and double-stranded RNases (33). However, the annealing products were not structurally characterized in further detail, and their possible role as intermediate forms between the two major forms of the IRES, LRA and stem-loop VI, was not determined.

The high concentration of drug needed to observe the *in vitro* results deserves further consideration. The commercial RNase III used in this study derives from a recombinant protein that has a focus on dsRNA cleavage, rather than processing a long ssRNA with a few specific sites for cleavage, which is the particular case in HCV RNA. This is open to the possibility of contamination by traces of conventional RNases, which can cleave ssRNA, or the possibility that the recombinant enzyme acquires a subtly broadened specificity (32). To avoid these potential problems, some of which may differ between different RNase III batches, we included a large quantity of highly purified yeast tRNA (58 μM) in our RNase III reactions, which served to protect our RNA from undesired activities accompanying RNase III (32). We described the reaction conditions previously (32), and in this study, we refer to them as standard conditions. The conditions associated with our study are that tRNA binds aminoglycosides from different families and with an affinity of ∼100 to 200 μM (68). Thus, *in vitro* RNase III reactions were performed at an HCV RNA/tRNA molar ratio of 6 × 10^−4^ μM HCV RNA/58 μM yeast tRNA; this implies that drug inhibition is restricted to strong RNA binders, which are clearly able to compete with this high concentration of tRNA.

Detecting specific RNA structure-dependent cleavage inhibition by the drug not only provides an opportunity to interact specifically and alter the IRES structure between the two conformations (closed and open), which can help when studying the multifunctionality of this part of the viral genome, but it also gives us a potential therapeutic target that can eventually be studied to determine HCV's ability to undergo selective inhibition when grown in a cell. Since Geneticin, unlike all other aminoglycosides, is readily taken in by cells, we tested the effect of the compound on viral growth inside cells.

In the present study, we demonstrated that Geneticin selectively inhibits HCV Jc1 proliferation in Huh7.5 cells, as well as the expression of the HCV NS3 protein (with an EC_50_ of about 2 μg/ml). This is also consistent with Geneticin's capacity to dramatically block the release of HCV genomic RNA and the spread of viral infection. Geneticin-dependent inhibition of HCV Jc1 NS3-positive plaque formation decreased both plaque number and size, demonstrating inhibition of both replication and viral spread. The selectivity index (50% cytotoxic concentration [CC_50_]/EC_50_) of Geneticin's anti-HCV activity in Huh7.5 cells was determined to be about 265, demonstrating that the mechanism of Geneticin's antiviral activity is selective and differs from its toxic effects.

Geneticin's antiviral molecular mechanism remains unclear. To date, Geneticin has shown antiviral activity against different, but not all, RNA viruses in a variety of cell lines (10, 11), suggesting that the Geneticin-mediated antiviral mechanism does not depend on the cell type. Therefore, the antiviral activity of Geneticin is probably virus specific. In this study, we have shown that Geneticin can interact directly with the RNA switch motif in HCV RNA, supporting the idea that using aminoglycosides to target conserved regions of viral RNA has therapeutic potential. Furthermore, the interaction between Geneticin and the HCV RNA switch provides the first concrete evidence that the RNA switch within HCV RNA is important for the life cycle of HCV. Therefore, further exploration of Geneticin virus selectivity and comparison of the antiviral mechanisms of different aminoglycosides with respect to HCV are necessary.

The next phase of this work will involve experimental determination of the specific binding site for Geneticin using mutants generated *in vitro* and by determining the resistance of these virus mutants to the drug *in vivo*. Nevertheless, the main role of the drug activity *in vivo* is a not a simple issue. The structures involved in RNA switching, controlled by Geneticin, participate in several essential viral functions. The 5′-proximal region flanking the IRES has, quite convincingly, been observed to participate in translation inhibition, viral-RNA stabilization, and RNA replication. The 3′ IRES flanking sequence is thought to participate in replication and translation. Hence, when proximal and distal regions are combined through switchable long-distance annealing, the role associated with both of these regions might cover three areas of the virus' biology.
